# Sound symbolic congruency detection in humans but not in great apes

**DOI:** 10.1038/s41598-019-49101-4

**Published:** 2019-09-03

**Authors:** Konstantina Margiotoudi, Matthias Allritz, Manuel Bohn, Friedemann Pulvermüller

**Affiliations:** 1 Brain Language Laboratory, Department of Philosophy and Humanities, WE4, Freie Universität Berlin, 14195 Berlin, Germany; 2 Berlin School of Mind and Brain, Humboldt Universität zu Berlin, 10099 Berlin, Germany; 3 School of Psychology & Neuroscience, University of St. Andrews, St. Andrews, Fife UK; 4 Leipziger Forschungszentrum für frühkindliche Entwicklung, Universität Leipzig, Leipzig, Germany; 5 Department of Psychology, Stanford University, Stanford, USA; 6 Cluster of Excellence “Matters of Activity”, Humboldt Universität zu Berlin, 10099 Berlin, Germany; 7 Einstein Center for Neurosciences Berlin, 10117 Berlin, Germany

**Keywords:** Language, Human behaviour

## Abstract

Theories on the evolution of language highlight iconicity as one of the unique features of human language. One important manifestation of iconicity is sound symbolism, the intrinsic relationship between meaningless speech sounds and visual shapes, as exemplified by the famous correspondences between the pseudowords ‘maluma’ vs. ‘takete’ and abstract curved and angular shapes. Although sound symbolism has been studied extensively in humans including young children and infants, it has never been investigated in non-human primates lacking language. In the present study, we administered the classic “takete-maluma” paradigm in both humans (N = 24 and N = 31) and great apes (N = 8). In a forced choice matching task, humans but not great apes, showed crossmodal sound symbolic congruency effects, whereby effects were more pronounced for shape selections following round-sounding primes than following edgy-sounding primes. These results suggest that the ability to detect sound symbolic correspondences is the outcome of a phylogenetic process, whose underlying emerging mechanism may be relevant to symbolic ability more generally.

## Introduction

There has been a long debate in semantics as to whether the relationship between form and meaning of a sign is entirely arbitrary or not^1,2^. A classic example of non-arbitrariness in human language is sound symbolism. Sound symbolism describes the phenomenon that humans match pronouncable but meaningless pseudowords to specific visual shapes. Köhler^3^, who discovered sound symbolism, had reporting that the pseudoword ‘maluma’ was judged to be a good match to a curved shape whereas the pseudoword ‘takete’ was judged as better match to an angular shape. Instead of “sound symbolism”, other terms have been used, for example “phonetic symbolism”^4^ or “crossmodal iconicity”^5^.

A number of studies have documented sound-meaning mappings in speakers of a broad range of languages^6^, including South East Asian languages^7^, African languages^8^, Balto-Finnic^9^ and Indo-European^10^, thus ruling out language specificity as a possible factor. Although cross-modal sound symbolic relationships replicate across a wide range of experiments in human adults or children with variable language backgrounds using different stimuli, it still appears as a mystery why a majority of human subjects agree that certain speech items sound ‘rounder’ or ‘edgier’, and why certain visual and acoustic stimuli intuitively match with each other.

Sound symbolism has been claimed to facilitate language acquisition and development. For example, Maurer and colleagues^11^ have shown that 2.5 years old children matched oral sounds to shapes. Other studies tested whether sound symbolism facilitates verb learning and found positive evidence in 25-month-old Japanese^12^ and 3-years old English children^13^. In both studies, children learned novel verbs that sound-symbolically matched or did not match different actions. Based on their findings, children performed better on generalizing the novel verbs to the same actions but in different contexts (e.g. different actor performing the action), when the novel learned verbs sound-symbolically matched the described action during the learning phase. In contrast to these results from children already knowing some language, evidence for sound symbolic matching in preverbal infants is less conclusive. A sequential looking time study found that 4-month-old infants looked longer at incongruent correspondences between shape and sound than to congruent ones^14^. However, Fort and colleagues^15^ found no such evidence in 5 and 6-months old infants tested in a preferential looking paradigm. A recent meta analysis^16^ concluded that it is still unclear whether preverbal infants are capable of sound symbolic matching. Hence the sensitivity to sound symbolism in early life is an open issue.

Research in nonhuman animals has investigated the understanding of sound-image correspondence for familiar categories (e.g. vocalizations vs. faces of conspecifics, or human speech and human faces; e.g.^17–22^; see^23^ for a review). Animal research and specifically research in non-human primates has not directly addressed the question of abstract sound-shape correspondences. However, Ludwig and colleagues tested crossmodal correspondences between luminance and pitch in great apes^24^. In this study, 6 chimpanzees were trained to perform a speeded classification paradigm of squares with ‘high’ or ‘low’ luminance. During the testing phase, chimpanzees (as well as a human control group) performed the same task again, however now with ‘high’ - or ‘low’ -pitched sound co-presented. Chimpanzees, like humans, performed better when a congruent sound was presented (high-pitched sound with high-luminance square and low-pitched sound for low-luminance square) than an incongruent one. This finding suggests a general ability for cross-modality matching in great apes.

A number of recent studies tested production^25^ and comprehension^26,27^ of iconic gestures in chimpanzees and children. A study by Grosse *et al*.^25^ examined whether chimpanzees and 2–3 years old children use iconic gestures to instruct a human experimenter on how to use an apparatus. Chimpanzees, unlike human children, did not produce iconic gestures to instruct the human experimenter. In a similar vein, Bohn *et al*.^26^ tested comprehension of iconic gestures in chimpanzees and 4-year old children. In this experiment, the experimenter used either iconic or arbitrary gestures in order to inform the subject about the location of a reward. In contrast to children, chimpanzees showed no spontaneous comprehension of iconic or arbitrary gestures. A follow-up study also found no spontaneous comprehension when gestures were enriched with iconic sounds and preceded by a communicative training^27^. However, in the initial study, apes learned to associate iconic gestures with a specific location faster compared to arbitrary gestures. According to the authors, apes failed to spontaneously comprehend the gesture because they did not perceive it as communicative. Associative learning of gesture - location correspondence was enhanced in the iconic condition because seeing the gesture shifted apes’ attention to the corresponding apparatus by triggering a memory representation of the bodily movement, from which the gesture was derived, that was used to operate the apparatus. This evidence might be taken as a hint that apes have at least some tendency toward correctly interpreting at least some iconic manual gestures, thus raising the possibility that also other forms of iconicity may be available to them, which may or may not include sound symbolic congruency detection and matching.

Few hypotheses address the mechanistic cause of sound symbolic mappings. For example, Ramachandra and Hubbard^28^ proposed the “synaesthetic account of sound symbolism”, which is based on putatively innate knowledge about correspondences between visual shapes and phonemic inflections. According to the authors the mechanism behind this effect has an articulatory account. For example, the sharp edges of a spiky shape mimic the sharp phonemic inflections and the sharp movement trajectory of the tongue on the palate when uttering the pseudoword “kiki”. The authors see such “synesthetic correspondence” as important in the emergence of language.

The hypothesis that language and sound symbolic processing are intrinsically related to each other raises the question whether both of these effects are only present in humans, but not in non-human primates. In fact, brain organization in great apes, and in particular chimpanzees, shows reasonable similarity to humans, although there are, no doubt, anatomical differences, which have their correlate at the highest functional level in the presence and absence (or great limitation) of language.

Neuroanatomical studies have shown that a major difference setting apart humans from their closest relatives, chimpanzees, lies in the much stronger and richer development of a neuroanatomical fiber bundle called the arcuate fasciculus (AF)^29,30^. This fiber bundle connects the anterior and posterior language areas in frontal and superior temporal cortex with each other, but also interlinks the ventral visual stream of object related form and color processing with the latter^31^. The AF is known to be important for interlinking information about articulatory movements with that about acoustic signals produced by the articulations, thereby laying the ground for abstract phonological representations that span across modalities^32–34^. Similarly, the AF may play a main role in linking letters to sounds, and it is likely that it stores other types of cross-modal symbolic relationships too. Experimental evidence has shown functional relationships of the AF in humans with their ability to store verbal materials (verbal working memory, VWM) and its general relevance for language processing^35^. We hypothesize that a strongly developed human-like AF is also involved in, and necessary for, the kind of abstract cross-modal information linkage required for sound symbolism. This position predicts a fundamental difference in sound symbolic ability between humans and apes which parallels their difference in language capacity.

It is evident that apes can differentiate forms and shapes^36,37^ and some research also indicates that they can perceive differences in human speech^38–41^. Considering these two abilities, the present study aims to explore whether our closest living relatives process sound symbolic mappings between shapes and sounds. We attempted to replicate existing findings in sound symbolic matchings in human adults using a two-alternative forced choice (2AFC) task, and performed a similar ape-compatible 2AFC task with a group of touchscreen trained great apes to investigate if any sound symbolic congruency effect would be present.

## Experiment 1

### Method

#### Subjects

Twenty-four healthy right-handed adults (14 females, age M = 25.87, SD = 5.08) participated in the study. The subjects were native speakers of different languages (11 German, 3 Greek, 2 Italian, 2 Spanish, 1 French, 1 Bulgarian, 1 Russian, 1 Urdu, 1 Kurdish, 1 Afrikaans). Two of the subjects where bilinguals, one speaking Greek and Albanian, one Afrikaans and English. All subjects had normal hearing and normal or corrected to normal vision. Subjects were recruited from announcements at the Freie Universität Berlin. All methods of the study were approved by the Ethics Committee of the Charité Universitätsmedizin, Campus Benjamin Franklin, Berlin and were performed in accordance with their guidelines and regulations. All subjects provided written informed consent prior to the participation to the study and received 10 euros for their participation.

#### Material and procedure

Angular or curved shapes were created in Power Point with the freeform tool and edited on GNU Image Manipulation Program (The Gnu Image Manipulation Program Development Team, 2010; www.gimp.org). Each shape was black (RGB 0,0,0) and 350 × 350 pixels in size. For the selection of the final shapes, a separate group of subjects (N = 110, recruited online via mailing lists) judged how angular or curved each shape was on a 7-point likert scale, ranging from 1-angular to 7-curved. We selected the 12 most angular (M = 2.00, SD = 0.34) and curved (M = 5.32, SD = 0.58) shapes, respectively (see Supplementary Table S1). For all selected shapes, the sum of responses in the range 1–3 (angular) or in the range 5–7 (curved) was three times higher, than the number of responses for 4-point (neutral) or for the other half of the scale. Auditory stimuli were created based on a previous studies regarding the role of consonants^10,42^ and vowels^11^ in sound symbolism. We used combination of vowels and consonants that have been previously reported sounding more ‘round’ or ‘sharp’ respectively. We created trisyllabic or disyllabic pseudowords with combinations from the following letters: the front vowels /i/ and /e/, the back vowels /o/ and /u/, the fricatives /z/, /s/ and /f/, the voiceless plosives /p/, /t/ and /k/, the nasals /m/ and /n/ and the voiced plosives /g/, /d/ and /b/. A separate group of subjects (N = 92, again recruited via online mailing lists) rated these pseudowords on a 7-point likert scale, ranging from 1-‘sharp’ to 7-‘round’ sound. The ‘sharperst’ pseudowords had the combination of the front vowels /i/ and /e/, the fricatives /z/ and /s/ and /f/, and the voiceless plosives /p/ and /k/ (M = 2.8, SD = 0.22), whereas the ‘roundest’ words were combinations of the back vowels /o/ and /u/, the nasals /m/ and/n/ and the voiced plosives /g/ and /d/ (M = 5.4, SD = 0.34). For the final experiment, we decided to use disyllabic pseudowords with a consonant-vowel-consonant-vowel (CiViCiVi) structure, for example “lolo” or “kiki”, based on the combinations of consonants and vowels determined by the online questionnaire. We included 10 ‘sharp’ and 10 ‘round’ pseudowords for each category. The auditory stimuli were recorded in a soundproof booth by a female native Greek speaker in Audacity (2.0.3) (http://audacityteam.org/) and afterwards normalized for amplitude. For the list with the final stimuli (see Supplementary Table S2).

**Figure 1:**
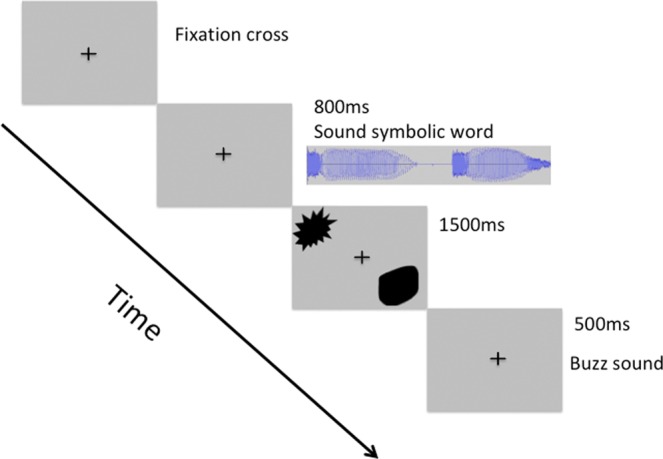
Schematic representation of experimental design of the two-alternative forced choice (2AFC) task applied in humans.

Both humans and apes performed a 2AFC task. Evidence suggests that apes are able to perceive differences in abstract forms and shapes presented to them on computer screens^36,37^. Furthermore, it has been shown that under specific circumstances, apes also perceive differences between human speech utterances^38–40^. Each trial started with the presentation of a fixation cross for 500 ms followed by the presentation of an auditory stimulus for 800 ms. Next, the two target shapes always one angular and one curved appeared diagonally, on the screen from upper left to bottom right or reverse. These stayed on screen for 1500 ms; during this time, responses were collected. Every trial ended with the presentation of a ‘buzz’ sound lasting 500 ms (see Fig. 1). All slides were presented on a grey background (RGB 192,192,192). The experiment was divided into 3 blocks (80 trials each) separated by two pauses in between. In each block, 10 specific combinations assembled from the selected 12 shapes and 10 sounds were used. These repeated within blocks, but were different between blocks. All trials were randomized within each block.

Human subjects sat in a dimly lit room in front of a 23 in. LCD monitor (screen refresh rate 75 Hz; screen resolution 1280 × 1024). The auditory stimuli were presented via two Logitech speakers (Model NO: Z130) located at each side of the screen. Responses were recorded via two-button press on a Serial Response BoxTM (SRBox, Psychology Software Tools, Inc). The experiment was designed in E-Prime 2.0.8.90 software (Psychology Software Tools, Inc., Pittsburg, PA, USA). Before the initiation of the experiment, subjects received the following written instructions: “During the experiment two pictures will appear, one low and one high on your screen, presented after a sound. Please choose one of the two pictures that matches the sound you hear”. No specific instructions were given to the participants regarding speed or accuracy. By the end of the experiment subjects completed a computer-based questionnaire about their strategies on shape selection and on their previous knowledge on sound symbolism.

#### Data analysis

For all analyses, trials with reaction times greater than 1500 ms or non-response were excluded. To check if subjects’ selection of shapes was influenced by the sound, we performed Wilcoxon signed-rank tests to compare the number of congruent (sound symbolic) responses against chance level. In order to check if performance was further influenced by other variables, in an exploratory analysis, we fitted a generalized linear mixed model (GLMM) with a binomial error structure. As analysis tool, R version 3.4.3 was used including the package lme4^43^. The dependent variable was congruency that is whether the shape of the selected stimulus matched the shape of the primed sound. We included word type (‘sharp’ vs ‘round’) and trial number as fixed effects. We used a maximal random effect structure with random intercepts for subject, word and for the combinations of the presented shapes and random slopes for each trial nested within these random effects. We used the likelihood ratio test (LRT) to check if the predictor variables improved the fit of the model; these were calculated by comparing the full model to a reduced model that included all terms except for the fixed effect term in question. Chi square and p-values were computed using the function drop1 from the R package lme4. In addition, we compared individual proportions of incongruent responses for ‘round’ and ‘sharp’ words using Wilcoxon signed rank test as well as the individual proportions of congruent responses for each pseudoword category against chance level. Finally, we calculated the proportion of times each subject chose a curved or angular shape (independent of the previous accoustic stimulus) and performed a Wilcoxon signed rank test.

### Results

We excluded 3.1% of the trials obtained from humans, because reaction times where greater than 1500 ms or non-response was given. Humans showed a significant preference for image choices with sound symbolic correspondence to the preceding sounds (V = 296; p = 0.001; see Fig. 3). An average of 71.33% or congruent shape choices contrasted with only 28.67% incongruent responses. In addition, the predictor variable of word type significantly improved the model (*χ*^2^(1) = 27.30, p = 0.001). Specifically, there were more congruent responses for ‘round’ than for ‘sharp’ pseudowords (see Fig. 4a). Incongruent responses were primarily seen for ‘sharp’ words being classified as ‘round’ (6.16% incongruent ‘round’ responses vs. 22.16% incongruent ‘sharp’ responses (V = 300, p = 0.001) (see Supplementary Fig. S1). Corresponding result was revealed by the analysis on the proportion of congruent responses for each pseudoword category against chance, with ‘sharp’ pseudowords perhaps showing a tendency but not significantly exceeding chance level (V = 188, p = 0.14) and ‘round’ congruent responses clearly ending up above chance (V = 300, p = 0.001). Humans selected curved shapes in 66.8% of cases, significantly more often than angular shapes (V = 0, p = 0.001) (see Supplementary Fig. S3). Figure 3 shows that the range of human performance varied widely from chance to 71.33% congruent responses. Closer examination of the individual subjects’ behavior and performance was conducted to assess whether all subjects performed the task as instructed. It turned out after the experiment when filling out the post-experiment questionnaire, that one individual’s understanding of the English language – the language in which instruction were given – was very limited. Three other participants showed an extreme preference for curved shapes, which they chose over 80% of the trials. This is quite unusual behavior (also not paralleled by any of our apes) and we therefore excluded these four ill-behaving subjects. Their results are highlighted in pink in Fig. 3. Please note that any sound congruency effects in these subjects’ responses were absent, with performance approximating chance. A new analysis conducted on the data from the remaining 20 individuals confirmed the presence for sound symbolic congruent over incongruent responses (V = 210; p = 0.001). The comparison of individual proportion of incongruent responses for the two pseudoword categories remained significant (5.76% incongruent ‘round’ responses vs. 18.42% incongruent ‘sharp’ responses (V = 210, p = 0.001). On the other hand, the analysis on the proportion of congruent responses for each pseudoword category against chance revealed that both ‘sharp’ pseudowords (V = 173, p = 0.004) and ‘round’ (V = 210, p = 0.001) exceeded chance levels.

**Figure 2:**
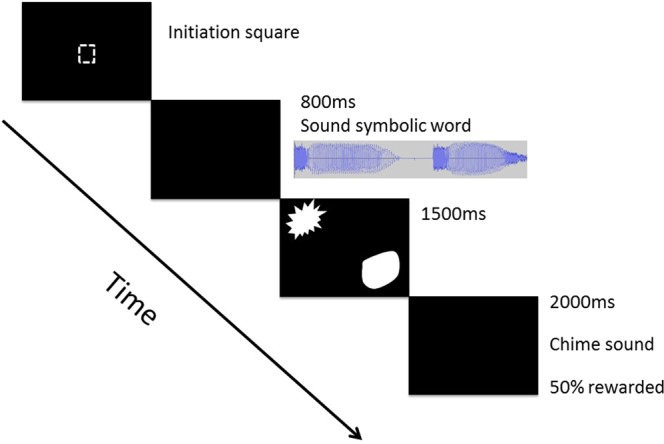
Schematic representation of experimental design of the two-alternative forced choice (2AFC) task applied in apes.

**Figure 3:**
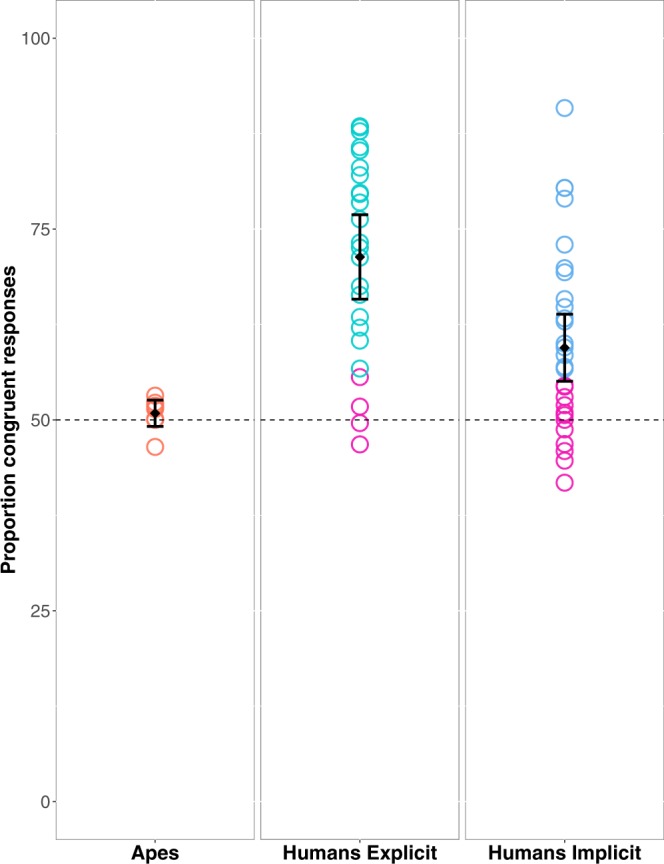
Percentage of sound symbolic congruent responses for apes and for humans performing on the explicit and the implicit 2AFC task, quantified as the proportion of times each individual matched a ‘sharp’ sound to an angular shape or a ‘round’ sound to a curved shape. Orange, cyan and blue circles show the percentage of congruent responses for individual apes and humans for the explicit and implicit instructions separately. Pink circles represent the human subjects that reached the ape performance. Black diamonds represent the average responses for each species and the whiskers show 95% confidence intervals (CIs). The dashed line at 50% shows chance-level performance.

**Figure 4:**
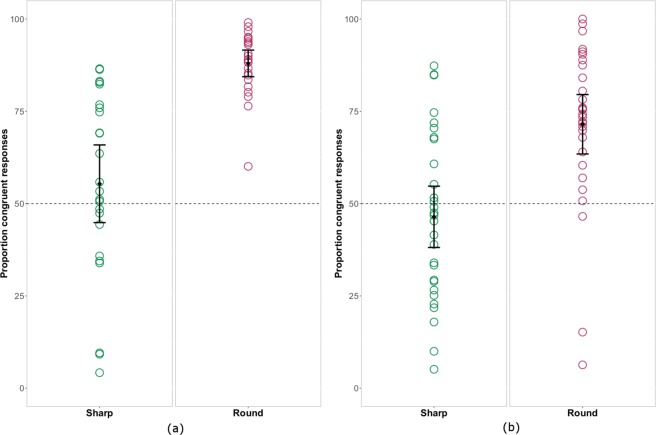
(**a**) Proportion of sound symbolic congruent responses in humans for the two pseudoword categories in the explicit 2AFC task. Green and maroon circles show the percentage of congruent responses for each individual for sharp and round pseudowords separately. Black diamonds represent the average responses for each pseudoword category and whiskers show 95% confidence intervals (CIs). The dashed line at 50% shows chance-level performance. (**b**) Proportion of sound symbolic congruent responses in humans for the two pseudoword categories in the implicit 2AFC task. Green and maroon circles show the percentage of congruent responses for each individual for sharp and round pseudowords separately. Black diamonds represent the average responses for each pseudoword category and whiskers show 95% confidence intervals (CIs). The dashed line at 50% shows chance-level performance.

## Experiment 2

### Method

#### Subjects

Six chimpanzees (3 females) and two gorillas (2 females) (age M = 20.75, SD = 13.18) housed at the Wolfgang Köhler Primate Research Center (WKPC) at Leipzig Zoo, Germany, participated in the study. Apes were never food or water deprived. Food rewards from the study were given in addition to their regular diet. Participation was voluntary and apes could abort the experiment at any time. The study was approved by an internal ethics committee at the Max Planck Institute for Evolutionary Anthropology, Leipzig, Germany. Research was non-invasive and strictly adhered to the legal requirements of Germany. Animal husbandry and research complied with the European Association of Zoos and Aquaria (EAZA) Minimum Standards for the Accommodation and Care of Animals in Zoos and Aquaria and the World Association of Zoos and Aquariums (WAZA) Ethical Guidelines for the Conduct of Research on Animals by Zoos and Aquariums.

#### Material and procedure

The study was conducted in the apes’ familiar observation or sleeping rooms. We installed an infrared touchscreen (Nexio NIB-190B infrared touch screen) outside to the testing cage. The screen was connected to a 19 in. computer monitor with a resolution of 1280 × 1024 (aspect ratio 5:4) fixed behind the touchscreen. Sound was played through two loudspeakers placed on the floor next to each side of the monitor, 2 Logitech speakers (Model No: X-120) for the chimpanzees and 2 Logitech speakers (Model No: x-140) for the gorillas. The stimuli were the same as the ones used for humans. We made the following adjustments to the setup: The background of the slides was black (RGB 0,0,0) and the shapes were white (RGB 255, 255, 255) in order to have high contrast and to maintain the attention of the apes (see Fig. 2).

The ape experiment was designed to be as similar as possible to the human one. However, some modifications were necessary to accommodate between-species differences and especially to replace the verbal instruction given to humans by a training procedure with direct reinforcement. Every trial started with an initiation symbol that the ape had to touch in order to start the trial. This self-initiation procedure has been used before to ensure that the apes are attentive at the beginning of the trial^44,45^. If the ape did not engage with the touchscreen for a certain amount of time, the session was terminated prematurely. After touching the initiation square, a disyllabic pseudoword was presented for 800 ms followed by the presentation of two shapes diagonally. The response time window was the same as for humans (1500 ms). The last slide was the reward or no reward slide, namely a black blank slide that remained on the screen for 2000 ms, which was either combined with a reward-announcing ‘chime’ sound (Windows XP Default.wav) or not. Within these 2000 ms after the ‘chime’ sound, a reward (a piece of apple) was delivered. We used the ‘chime’ sound, as it has been previously used to announce the delivery of the food reward in the same apes^45^. There was a 50% chance for a given trial to be followed by a reward-announcing sound and actual reward. This random rewarding procedure was implemented to maintain the subject’s motivation to continue partaking. Note however, that the type of response, whether an angular or curved shape was selected, did not influence the likelihood of the reward, thus excluding any bias toward ‘congruent’ or ‘incongruent’ responses.

To familiarize apes with the 2AFC task, they had to perform up to three habituation sessions. A habituation session consisted of 80 trials in which different combinations of disyllabic pseudowords, irrelevant to the experiment were presented along with random combinations of shapes used in the experiment. The apes were rewarded every time they selected one of the two shapes followed by the positive ‘chime’ sound. The purpose of the habituation sessions was to assure that the apes would not be surprised or midly agitated by the sound stimuli and they would touch one of the two shapes within the specific response time window. Five chimpanzees completed one habituation session, one completed two and both gorillas completed three. In order to move from the habituation to the testing phase the ape had to make a selection 80% of the times within the specific response time window and look at the touchscreen during every trial.

The experiment consisted of 6 blocks of 80 trials each. As in Experiment 1 the combinations of sounds and shapes differed across the 3 blocks. These 3 blocks were the same as those used with humans; with apes, they were repeated to yield the overall 6 blocks. The same sound-shapes trial was not presented in more than one block across the first 3 blocks. The order of trials was randomized within each block. Apes were tested in one block per day to avoid any habituation effects.

#### Data analysis

The analyses were similar to the ones conducted for Experiment 1. For apes, we excluded 13.88% trials with responses above 1500 ms or non-responses. The GLMM model for apes included as dependent variable congruency, that is whether the shape of the selected stimulus matched the shape of the prime sound, and as fixed effects word type, trial and block. We used a maximal random effect structure with random intercepts for participant, word and the trial-specific combination of shapes, as well as random slopes for trial and block. In order to explore any effect of the reward schedule on the performance of apes, we fitted generalized linear mixed models. In the first model, we included as dependent variable the shape category the apes selected (‘sharp’ or ‘round’) for each trial and as fixed effects the shape category selected in the previous trial if and only if this trial had been rewarded, as well as the fixed effects trial and block. We used a maximal random effect structure with random intercepts for participant and word, as well as random slopes for trial and block. The likelihood ratio test (LRT) was applied to check if the predictor variable improved the fit of the model; these were calculated by comparing the full model to a reduced model that included all terms except for the fixed effect term in question. Chi square and p-values were computed using the function drop1 from the R package lme4. Finally, we used Mann-Whitney U test to compare the congruency responses between Experiment 1 and 2.

### Results

Due to the small sample size of the two non-human primate species we could not make any statistical inferences on their performance separately. However a visual inspection of the results showed no difference in the performance of chimpanzees and gorillas. Numerically, both species performed similarly, with gorillas reaching 51.17% congruent responses and chimpanzees 50.75% (see Supplementary Fig. S2). Apes, showed no preference for sound symbolic correspondences (V = 21; p = 0.27) (see Fig. 3). There was also no significant difference between the full and the reduced model (*χ*^2^(1) = 2.28, p = 0.13), indicating that word type (round or sharp), block and trial, considered in conjunction, did not improve the predictive accuracy of the model. Moreover, they tended to have similar congruency effects for ‘sharp’ and ‘round’ words (27.16% incongruent ‘round’ responses vs. 22.07% incongruent ‘sharp’ responses, W = 20, p = 0.23) (see Supplementary Fig. S1). Furthermore, apes did not indicate also any bias towards selection of one of the two shape types (45.11% curved vs. 54.88% angular responses; W = 44, p = 0.23) (see Supplementary Fig. S3). The result of the reward analysis revealed that the subjects’ choices did not differ significantly depending on whether a reward on a preceding trial was received after touching a round vs. sharp image. Specifically, there was no significant difference between the full and the reduced model after (*χ*^2^(1) = 0.25, p = 0.61). Thus, in a trial by trial analysis, the shape selected and rewarded in a given trial did not affect the shape selected in the following trial. Comparing the result patterns between Experiment 1 and 2, it can be seen that apes and humans show almost non-overlapping distributions of sound-congruency effects (W = 175, p = 0.001). The four human subjects that performed at a level similar to apes were those with evidence for non-cooperative task performance; after their removal, the distributions were fully distinct. Calculating chi-square tests for each participant, including apes and humans, there was significant above-chance performance for 20 out of 24 human subjects but for none of the apes.

### Interim discussion

The results from Experiments 1 and 2 suggest that sound symbolic congruency effects are present in humans but not in great apes. However, before we will discuss this putative conclusion in detail, an obvious caveat of the preceding experiments needs to be taken into account. Human subjects were explicitly instructed to perform sound symbolic matchings, whereas apes were trained to respond to pairs of visual displays by selecting one, without any task instruction or other hint about the ‘desired’ outcome being given. This obvious difference and potential confound of the previous results was addressed in Experiment 3 where a new set of human subjects was tested without explicit task instruction hinting at the sound symbolic correspondences our research targets.

## Experiment 3

### Method

#### Subjects

Thirty-one healthy right-handed adults (17 females, age M = 25.35, SD = 3.56) participated in the study. The subjects were native speakers of different languages (11 German, 3 English, 3 Spanish, 2 Mandarin, 2 Greek, 2 French, 1 Bulgarian, 1 Italian, 1 Romanian, 1 Czech, 1 Polish, 1 Malaysian). Two of the subjects where bilinguals, one speaking English and Spanish, one Spanish and German. All subjects had normal hearing and normal or corrected to normal vision. Subjects were recruited from announcements at the Freie Universität Berlin. All methods of the study were approved by the Ethics Committee of the Charité Universitätsmedizin, Campus Benjamin Franklin, Berlin and were performed in accordance with their guidelines and regulations. All subjects provided written informed consent prior to the participation to the study and received 10 euros for their participation.

#### Material and procedure

In order to explore further any possible effect of the explicit instruction given in Experiment 1 on the performance of humans, we conducted an additional experiment in humans similar to Experiment 1. The materials were the same as in Experiment 1. The experimental design and procedure were also alike with the following modifications. We reduced the total number of trials into 2 blocks (80 trials each) separated by one pause in between. In each block, 10 specific combinations assembled from the selected 12 shapes and 10 sounds were used. These were repeated within blocks, but were different between blocks. No ‘buzz’ sound was presented at the end of each trial. All trials were randomized within each block. In addition we modified the written instructions given to the subjects before the initiation of the experiment.

To provide a social motivation for performing on the task, subjects were informed about their reimbursement before the experiment and they received the following written instructions: “During the experiment two pictures will appear, one low and one high on your screen, presented after a sound. Please choose one of the two pictures”. Note that this instruction lacks any information about any type of matching to be performed. If such matching is observed in this experiment’s context, it cannot therefore be driven by instruction. Furthermore, the instruction did not specify response speed or accuracy. After the experiment, subjects completed a computer-based questionnaire about their strategies on shape selection and on their previous knowledge on sound symbolism.

#### Data analysis

For all analyses, trials with reaction times greater than 1500 ms or non-response were excluded. Data analyses were the same as in Experiment 1.

### Results

We excluded 2.5% of the trials, because reaction times where greater than 1500 ms or non-response was given. As in Experiment 1 humans showed a significant preference for image choices with sound symbolic correspondence to the preceding sounds (V = 367; p = 0.001). An average of 59.44% of congruent responses contrasted with 40.56% incongruent responses. However, the performance of more subjects dropped to chance level compared to Experiment 1 (see Fig. 3). The predictor variable of word type significantly improved the model (*χ*^2^(1) = 30.05, p = 0.001). In accordance with Experiment 1, there were more incongruent responses for ‘sharp’ than for ‘round’ pseudowords (see Fig. 4b). Once again, the incongruent responses were primarily seen for ‘sharp’ words being classified as ‘round’ (13.65% incongruent ‘round’ responses vs. 26.89% incongruent ‘sharp’ responses (V = 433, p = 0.001) (see Supplementary Fig. S1). A corresponding result emerged from the analysis of the proportion of congruent responses for each pseudoword category against chance, with ‘sharp’ pseudowords not exceeding chance level (V = 200, p = 0.82) and ‘round’ congruent responses being significantly above chance (V = 446, p = 0.001). As in Experiment 1 humans selected curved shapes in 63.21% of cases, significantly more often than angular shapes (V = 60, p = 0.001) (see Supplementary Fig. S3). Calculating chi-square tests for each participant there was significant above-chance performance for 18 out of 31 human subjects. The comparison between Experiment 2 and 3 revealed again that apes and humans show non-overlapping distributions of sound-congruency effects (W = 177, p = 0.03). In contrast, there was no significant difference in the performance of human subjects between Experiment 1 and 3 (W = 188, p = 0.99).

### Discussion

The present study used a 2AFC task to test whether humans and great apes spontaneously detect sound symbolic correspondences between abstract visual shapes and meaningless word-like combinations of speech sounds. Results indicate that humans’ forced choices of shapes were significantly biased by prime sounds towards selection of shapes that showed sound symbolic congruency with the primes, whereas great apes did not give evidence of any such sound symbolic congruency detection. In our human populations, this sound symbolic effect was mainly carried by ‘round sounding’ pseudowords. Whereas they may have tended to select angular shapes more frequently than curved ones after perceiving correspondingly ‘sharp sounding’ syllable combinations, only the opposite preference in favor of curved shapes was clearly manifest after ‘round sounding’ syllables. The same general result was obtained after explicit task instruction to “match shapes to sounds” (Experiment 1) and similarly when humans were given just a picture selection task instruction with the sound symbolic task aspects remaining fully implicit and opaque (Experiment 3). When humans were explicitly instructed to match pseudowords to shapes in Experiment 1, only four of them performed, similarly to all of the apes, at chance level, which suggests lack of task instruction understanding in these human individuals. However, performance dropped to chance level for 18 our of 31 human subjects in the ‘implicit’ Experiment 3, while still remaining significantly above chance at the level of group statistics.

There are obvious limitations in testing different species on tasks aiming at higher cognitive abilities such as cross-modal congruency processing. Although we chose a general task applicable to humans, chimpanzees and gorillas, namely the 2AFC, we had to introduce some modifications to adjust it to testing great apes. We will discuss these differences between the 2AFC tasks one by one. First, humans performed all testing blocks in one session, whereas apes performed one block per testing session, completing six sessions in total. Included in our main analysis, the predictor “block” did not modulate the apes’ performance, thus arguing against this difference being relevant for explaining between-species differences in performance. Second, humans registered their answers through a keyboard, whereas apes used a touchscreen; however, it is not obvious why humans should have responded differently in the Experiments 1 or 3, had they been using a touch screen. Concerning potential touch location biases that the apes may have shown, note that the position of the curved and angular shapes were balanced across trials so that such a bias could also not have influenced the results. It was critical to provide randomly delivered food rewards to the apes to train them for the task, to compensate for the impossibility to use verbal instruction, and in order to continuously motivate them and keep them engaged across testing. Indeed the 50% administration of food reward which was orthogonal to the task was efficient in that subjects were motivated to complete the data collection. Moreover, our reward analysis showed that the presence of the reward had no effect in shaping the choice of shape selection on a trial by trial basis in apes. Although humans received no reward in a trial by trial schedule, they were socially rewarded by monetary compensation, and they were made aware of such social reward before starting the experiment. Especially the social reward for performing choice responses to pictures (without further instruction) in Experiment 3 seems to us a reasonable match of the unspecific food reward our apes received.

The verbal instruction for humans in Experiment 1 to select a shape “that matches the sound” were reasonably efficient as well, although three out of 24 subjects did not follow them well. In order to exclude any possible effect of the explicit verbal instructions on humans’ performance in Experiment 1 and on explicitly paying attention to the pseudoword, we conducted Experiment 3. In this Experiment we performed a similar 2AFC task but with ‘implicit’ instructions. This means that participants were not instructed to pay attention to the sounds and they were not asked to select a shape that “matches the sound” as in Experiment 1. Instead, they were just instructed to select a shape with any sound symbolic aspects of the task remaining fully implicit and opaque to the participant. The results indicate that participants’ performance dropped compared to Experiment 1 and relatively more humans performed at chance level. However, and crucially, the different task instructions of Experiment 1 and 3 did not significantly alter the sound-symbolic performance pattern in humans.

In the future, it may be worthwhile to adopt a direct reinforcement paradigm to humans to potentially efficiently motivate consistently cooperative task performance in this species too. This could be done by using a food reward as with the apes, or, more conventionally, by providing the monetary reward piecemeal, on a trial by trial basis. However, it seems unlikely that such ‘reinforcement instruction’ may change the strong preference of human subjects for sound congruent responses as showed in Experiment 1 and 3. After all, social reward by reimbursement at the end of the experiment and possibly the self-reward resulting from the knowledge of acting as a cooperative experimental subject were already sufficient for allowing sound symbolic effects to emerge. Therefore, we do not believe that the remaining differences between the tasks applied in this study had a significant influence on the patterns of results obtained, and especially on the presence of the sound symbolic effect in humans.

### Crossmodal similarity processing in apes and humans

Even though the present study found no sound-shape correspondences in great apes, there is evidence that apes are sensitive to crossmodal mappings. As mentioned, Ludwig *et al*.^24^ showed that apes are able to process crossmodal correspondences between pitch and luminance, as they matched a high luminance stimulus to a high-pitched sound and a dark stimulus to a low-pitched sound. In a similar vein, another study showed that great apes can detect visual-auditory structural isomorphic patterns. In that study, two apes were trained to choose a symmetric visual sequence (e.g., two identical geometrical shapes separated by a a different third shape in between, for example ○□○)^46^. During the testing phase, the apes were presented with the trained symmetric visual pattern and with a non-symmetric pattern (e.g., two identical shapes followed or preceded by a third shape, for example ○○□). The visual presentation was preceded by an auditory pattern, either a symmetric (e.g., two high tones separated by a low tone) or non-symmetric one (e.g., two high tones preceded or followed by a low tone), which was either congruent or incongruent with the structure of the target trained symmetric visual sequence. When the presentation of the pattern was preceded by congruent auditory patterns, response latency to the symmetric visual patterns were shorter compared to when they were preceded by incongruent auditory patterns. The authors interpreted this result as evidence for crossmodal structure processing (priming) in chimpanzees.

In spite of these indications that apes can process cross-modality structural similarities, we did not find evidence for a matching between the visual and auditory domain for spoken pseudowords and contour stimuli. To what degree this lack of crossmodal interaction depends on the specific sound and visual stimuli used, their familiarity and specificity to the species, requires further study. Correspondences of the pitch-luminance type, could be explained by a common neuronal system of magnitude or energy (high vs low acoustic/light energy) across modalities, or simply by a ‘more or less’ in sensory neuronal activation^47^. The analogy between symmetric and asymmetric patterns across modalities can be formulized in terms of abstract structural patterns such as ‘ABA’ vs. ‘AAB’, and could be taken as evidence for abstract processes generalizing away from the individual stimuli and across modality-independent patterns. In contrast, the sound symbolic congruency between abstract shapes and pseudowords is not easily captured by comparable abstract rules or differences in magnitude or energy. If, for example, the articulatory account of sound symbolism is true, which posits that the maluma-takete effect stems from similarities between shapes and the tongue’s movement trajectory in the mouth, this may explain why apes in our study did not give evidence of processing this congruency that humans apparently perceived. Still, one may object that apes are well-capable of lip smacking and tongue clicking^48–50^, thus offering a potential basis for sensorimotor knowledge about sound symbolic correspondences, too. Based on this inconsistent picture, a question remains whether a similar congruency effect could be found in great apes, if picture and sound stimuli were more attuned to their species. This provides a possible reason why apes did not give evidence of processing such congruency. However, it still leaves open the important question which features of visual and acoustic materials make these items subject to sound symbolic congruency.

### Bias toward congruency for ‘round’

In both Experiment 1 and 3, humans gave more ‘congruent’ than ‘incongruent’ responses to ‘round’ than for ‘sharp’ pseudowords, and the predominance for congruent over incongruent responses was consistently significant only for the ‘round’ items. One may argue that the ‘round’ pseudowords we selected were more sound symbolic on average than the ‘sharp’ pseudowords, or that sound symbolic effects are generally carried by ‘round’ items only. However, the average scores on the ratings of the selected pseudowords could not support these hypotheses. The average “round- vs sharpness” ratings for the ‘round’ words were (M = 5.4, SD = 0.34), and the ‘sharp’ words (M = 2.8, SD = 0.22) were both equally far from the midpoint of the Likert scale (4.0) for’sharp’ words (V = 0, p = 0.001) and for round words (V = 190, p = 0.001). Even in the absence of a general bias in stimulus selection, a natural propensity in favor of congruent round responses was reported previously in the literature on sound symbolism^16,51^. Human children show an earlier and stronger sound symbolic effect for ‘round’ pseudowords^16^, but a much weaker effect for ‘sharp’ ones. A possible explanation for this general stronger effect of sound symbolism for ‘round’ pseudowords could be the natural tendency of people to prefer curved versus angular shapes, which has been reported earlier^52–54^. A strong preference for preferring round over sharp shapes was also clearly evident from human performance in the present experiments (Experiment 1: 66.8 vs. 33.2%; Experiment 3: 63.21 vs 36.79%). The observed difference in favor of ‘round’ pseudoword congruency responses and to the disadvantage of ‘sharp’ sounding words therefore appears to be the result of a response bias.

A preference for curved contours was found previously also in apes on a 2AFC task. Apes, in contrast to humans, preferred curved contours only when the presented items remained on screen until a response was registered, whereas humans preferred curved contours only after short presentation (80 ms) of the two item types^45^. Our present experiment with apes did not show any significant bias in favor or curved shapes. Contrasting with the human pattern, our apes tended to have similarly absent congruency effects for ‘sharp’ and ‘round’ words as well as similar probabilities of selecting curved and angular shapes (see Results for Experiment 2).

The lack of a preference for curvature in our study of eight apes stands in contrast to the findings by Munar *et al*.^45^, whose study of apes was conducted at the same facility and used a similar method. Their sample of apes was also of similar size (N = 9), four of whom participated in the present study. Differences in the types of stimuli that were used may explain why the original finding in apes was not replicated in this study. It is also possible that curvature preference may be too subtle to be detected reliably in small samples of apes, or it may be subject to procedural moderators.

### Sound symbolism is specific to humans

Our present data show that apes and human subjects produce clearly distinct response patterns of sound symbolic congruency effects. Whereas humans in both Experiments 1 and 3 consistently showed clear significant sound symbolic preferences at a population level, not a single ape did so. Even with non-cooperative subjects included in the human sample, there was a clearly significant between-species difference in the group analysis both between Experiment 1 and 2 (W = 175, p = 0.001) and between Experiment 2 and 3 (W = 177, p = 0.03). Although sound symbolic congruency detections in humans seemed to be more clearly apparent in Experiment 1 than in Experiment 3, there was no significant performance difference between their results (W = 188, p = 0.99). This robust difference may be related to the fundamental difference between the species in language ability. Humans share complex languages with large vocabularies and great combinatorial power as tool kit for communication whereas in apes, such a system is absent. We therefore suggest that sound symbolism may emerge from the same neuroanatomical connectivity that is also necessary and essential for the brain’s neuronal language circuits. If correct, this implies that human specificity of sound symbolism can be tracked down to anatomical differences between apes and humans revealed by comparative neuroanatomical data^30^. Comparative data suggest an expansion of the connectivity between perisylvian cortical areas involved in language in humans, which those in apes largely lack^30^. In particular, the AF, a left-lateralized long-distance corticocortical connection between inferior-frontal and posterior-temporal cortex, is relatively more strongly developed in humans^29,30^. Recent evidence from a computational model in human and non-human primates’ perisylvian language networks, showed better verbal working memory in humans^35^ explaining in part the weaker auditory memory documented in non-human primates^55,56^. The limited verbal working memory in apes prevents their word learning and phonological retrieval capacities, and these may also be fundamental for creating a repertoire of sound symbolic associations for social-interactive communication. It is also possible that, all other things being equal, humans exploit their AF connections when learning associating speech sounds/words and visual stimuli/abstract shapes. This is because the AF connects anterior language areas with both visual and auditory sites. The better developed AF in humans may therefore contribute to the possibility to store and process sound symbolic congruency, as it is crucial for building the brain’s language and phonological network. However, it is important to note that this is still a hypothesis. On its background, testing a language-trained ape for sound symbolic congruency processing appears as relevant. If anatomical connectivity structure determines sound symbolic processing ability, a language trained ape should still be unable to show it. In case sound symbolism is closely linked to language learning, we may predict sound symbolic congruency processing in apes with some linguistic competence.

To conclude, these results show no behavioral indication that great apes spontaneously perceive, recognize or infer cross-modal congruencies between speech sounds and abstract visual displays, whereas humans clearly show this type of crossmodal effect in both explicit and implicit 2AFC tasks. We suggest that the human specificity of sound symbolism may be linked to neuroanatomical differences between humans and apes in the connectivity structure of the perisylvian cortex which provides the basis for human language and possibly sound symbolic congruency too. Sound-shape mappings of this type might indeed have played a significant role in shaping human language.

## Supplementary information

Supplementary Material

## Data Availability

The datasets generated during and/or analysed during the current study are available from the corresponding author on reasonable request.
