# Supplementary material for: Sound symbolic congruency detection in humans but not in great apes
Source: Sci Rep. 2019 Sep 3;9:12705. doi: 10.1038/s41598-019-49101-4 (PMC6722092; doi:10.1038/s41598-019-49101-4)

Sound symbolic congruency detection in humans but not in great apes

Konstantina Margiotoudi[1,2*], Matthias Allritz[3], Manuel Bohn[4,5], and Friedemann Pulvermüller[1,2,6,7]

[1]Brain Language Laboratory, Department of Philosophy & Humanities, WE4, Freie Universität Berlin, 14195 Berlin, Germany
[2]Berlin School of Mind and Brain, Humboldt Universität zu Berlin, 10099 Berlin, Germany
[3]School of Psychology & Neuroscience, University of St. Andrews, St. Andrews, Fife, UK
[4]Leipziger Forschungszentrum für frühkindliche Entwicklung, Universität Leipzig
[5]Department of Psychology, Stanford University, Stanford, USA
[6] Cluster of Excellence "Matters of Activity", Humboldt Universität zu Berlin, 10099 Berlin, Germany
[7]Einstein Center for Neurosciences, Berlin 10117 Berlin, Germany

[*]konstantina.margiotoudi@fu-berlin.de

Supplementary Material

Table S1. Ratings of abstract shapes as obtained using an online questionnaire. Each shape selected for the study is listed with a running number, its mean rating (M) on a Likert scale (1- totally sharp; 7- totally round) and its standard deviations (SD).

Shapes	Nr	M	S.D
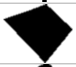	1	2.36	1.18
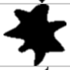	2	4.79	1.15
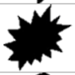	3	2.31	1.53
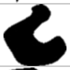	4	4.73	1.27
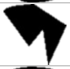	5	2.26	0.99
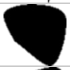	6	5.68	1.00
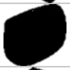	7	4.93	1.18
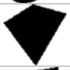	8	1.80	1.05
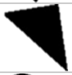	9	1.58	1.01
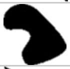	10	5.01	1.23
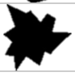	11	2.34	0.99

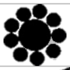	12	6.54	1.01
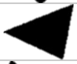	13	1.62	0.93
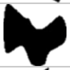	14	4.75	1.18
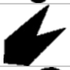	15	1.85	0.97
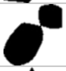	16	5.22	1.25
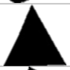	17	1.49	1.04
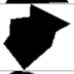	18	2.40	1.14
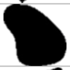	19	6.09	0.93
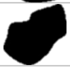	20	5.49	1.05

TableS2. Pseudoword stimuli for the two categories ‘sharp’ and ‘round’ sounding used in the experiments with humans and great apes.

**Sharp**	**Round**
kiki	nono
keke	nunu
sisi	momo
sese	mumu
fifi	lolo
fefe	lulu
zizi	dodo
zeze	dudu
pipi	gogo
pepe	gugu

FigureS1. Percentage of sound-symbolic congruent responses for each pseudoword obtained from apes and from humans for the explicit and implicit task. Green and maroon circles show the percentage of congruent responses for each ‘sharp’ and ‘round’ word separately. Black diamonds represent the average responses for each word category and the whiskers show 95% confidence intervals (CIs). The dashed line at 50% shows chance-level performance.

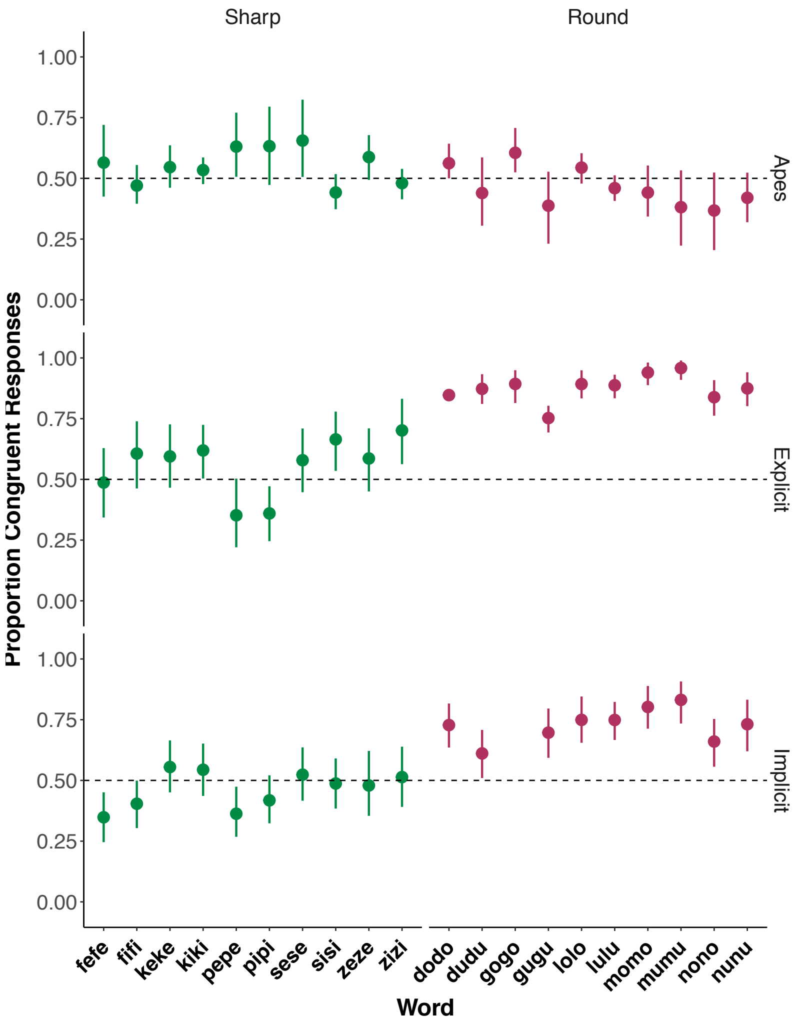

FigureS2. Percentage of sound-symbolic congruent responses in chimpanzees, gorillas and in humans tested in the explicit and implicit task, quantified as the proportion of times each individual matched a ‘sharp’ sound to an angular shape or a ‘round’ sound to a curved shape. Orange, purple and cyan and blue circles show the percentage of congruent responses for each. Black diamonds represent the average responses for each species and the whiskers show 95% confidence intervals (CIs). The dashed line at 50% shows chance-level performance.

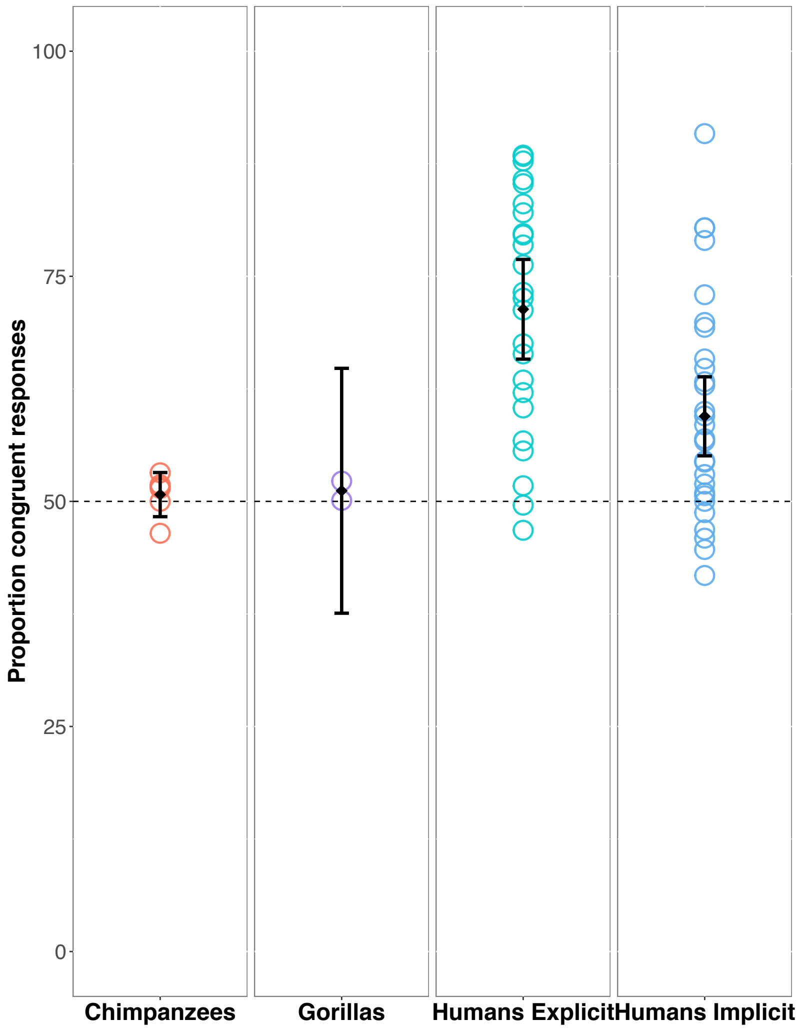

FigureS3. Proportion of curved shape selections in apes and in humans from both the explicit and implicit tasks separately. Orange, cyan and blue circles show the proportion of selecting a curved shape for individual apes and humans for the explicit and implicit task separately. Black diamonds represent the average responses for each species and the whiskers show 95% confidence intervals (CIs). The dashed line at 50% shows chance-level performance.

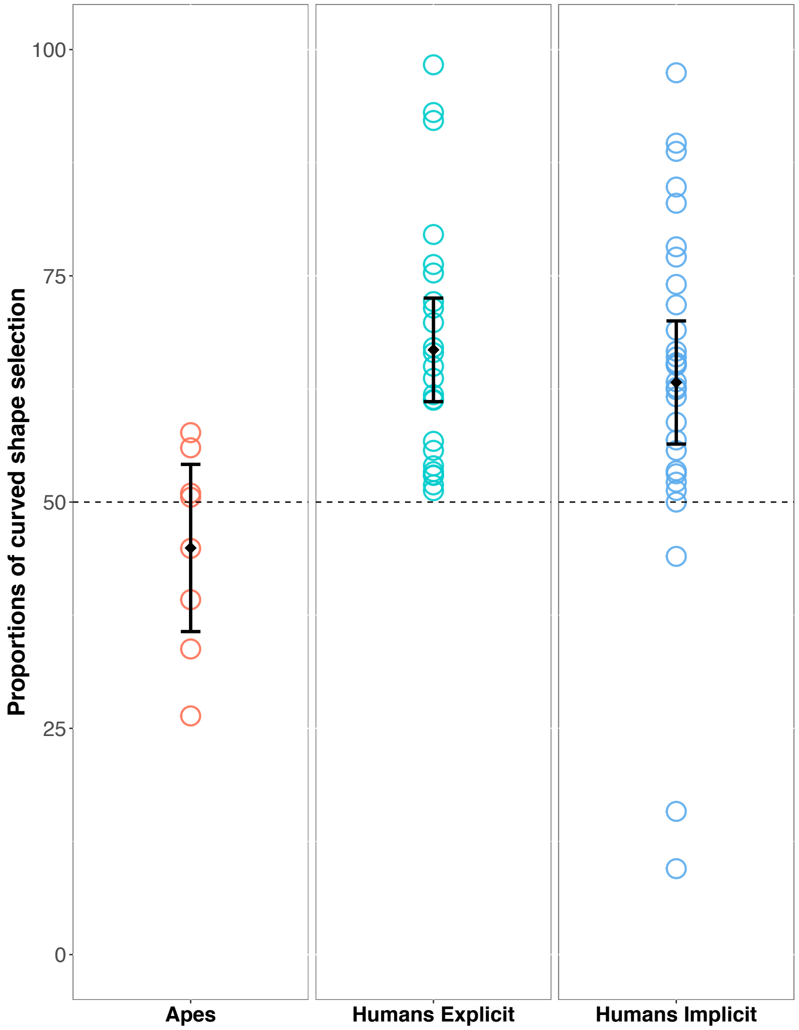

Supplement: Supplementary file 1 — Supplementary Material [file 41598_2019_49101_MOESM1_ESM.pdf]
